# Neuroimaging Predictors of Clinical Outcome in Acute Basilar Artery Occlusion

**DOI:** 10.3389/fneur.2017.00293

**Published:** 2017-06-19

**Authors:** Ravi Garg, José Biller

**Affiliations:** ^1^Department of Neurology, Stritch School of Medicine, Loyola University Chicago, Maywood, IL, United States

**Keywords:** hyperdense basilar artery, brain imaging, diffusion-weighted imaging, CT angiography, predictor, clinical outcome, basilar artery occlusion

## Abstract

Certain early neuroimaging findings are independent predictors of clinical outcome in acute basilar artery occlusion. These imaging findings may serve as important baseline imaging characteristics as well as subgroups in future randomized controlled trials. The purpose of this review is to review and compare early neuroimaging findings seen on non-invasive cranial imaging that predict clinical outcome in acute basilar artery occlusion.

## Introduction

Basilar artery occlusion is rare and may cause ischemia to brainstem, thalamic, and temporo-occipital structures. Clinical outcome following basilar artery occlusion may range from minor deficits to devastating neurological injury (1). The increased availability of early non-invasive cranial imaging has provided data to identify early neuroimaging findings that independently predict clinical outcome. These findings may be found on non-enhanced computed tomography (NECT), computed tomography angiogram source images (CTA-SIs), or diffusion-weighted magnetic resonance imaging (DWI). Additionally, early neuroimaging predictors may be primarily subdivided into those that evaluate for early ischemic changes, the extent of collateral flow, thrombus location, and thrombus burden. The hyperdense basilar artery (HDBA) sign is an exception to this subdivision.

## HDBA Sign

**Figure 1:**
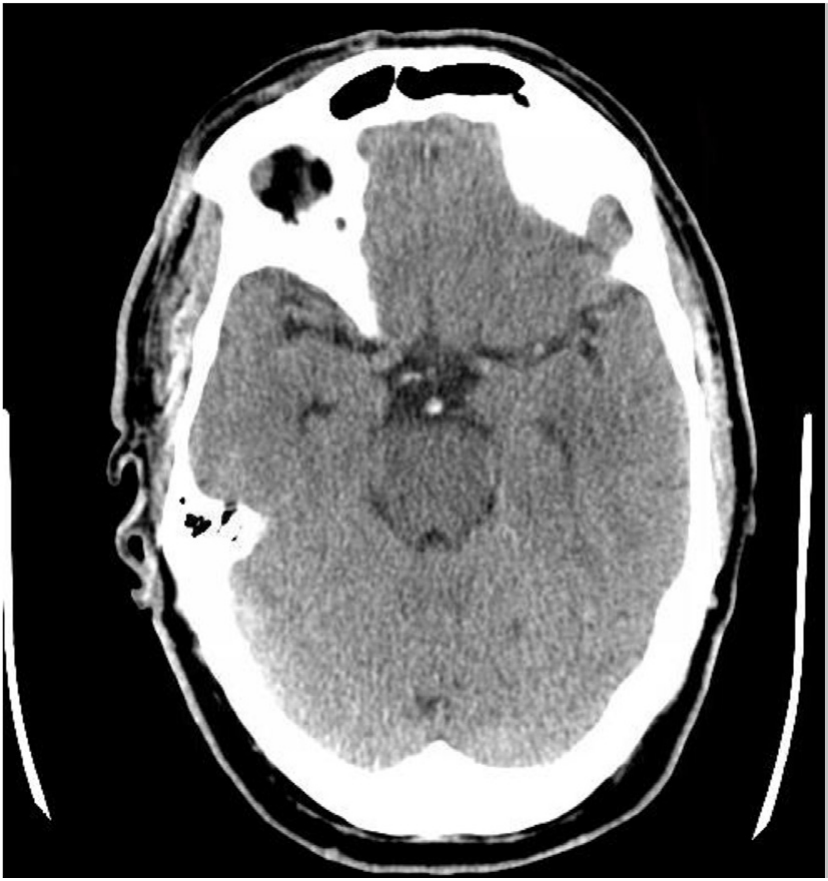
Hyperdense basilar artery (HDBA) sign. Axial non-enhanced computed tomography in a 56-year-old man who presented with unresponsiveness. Hyperdensity is visualized in the basilar artery consistent with a HDBA sign.

Similar to the hyperdense middle cerebral artery (MCA) sign, the HDBA sign on NECT (Figure 1) may be suggestive of acute thrombosis. In a study of clot composition in patients with MCA ischemic strokes treated with mechanical thrombectomy, patients with a hyperdense MCA sign on NECT had red blood cell predominant clots (2). This may be the most likely explanation for the presence of a HDBA sign in acute basilar artery occlusion. Goldmakher et al. examined the utility of the HDBA sign on NECT in detecting basilar artery thrombosis and predicting clinical outcome. They retrospectively reviewed 95 patients with documented posterior circulation infarction who received both a NECT and CTA on admission. Of these 95 patients, 14 (15%) had partial or complete occlusion of the basilar artery on CTA. Of these 14 patients, 10 (83%) had a HDBA sign on NECT. The presence of a HDBA sign on NECT was a significant independent predictor (OR, 5.6; 95% CI, 1.1–33.3) of a poor outcome defined as an modified Rankin score (mRS) of >2 at 6 months. The HDBA sign had a sensitivity of 71% and a specificity of 98% as a predictor of basilar artery occlusion (3).

Due to its retrospective nature, a limitation of this study was the elevated pretest probability of interpreting physicians. In a study by Connell et al., the presence of a HDBA sign had a lower sensitivity and specificity for acute basilar artery occlusion compared to that reported by Goldmakher et al. Connell et al. (4) also noted that quantitative measures of the basilar artery by measuring Hounsfield units may improve the diagnostic predictiveness of a HDBA sign on NECT. In another retrospective study, the presence of a HDBA sign predicted recanalization by intra-arterial thrombolytic therapy, but had no relationship with clinical outcome (5).

## Early Ischemic Changes

### Posterior Circulation Acute Stroke Prognosis Early CT Score (pc-ASPECTS) on CTA-SI

The Calgary Stroke Program developed a NECT-based scoring system to systematically assess early ischemic changes in anterior circulation ischemic infarctions (6). Similarly, Puetz et al. have developed a pc-ASPECTS to predict functional outcome in patients with basilar artery occlusion. The pc-ASPECTS is a 10-point scoring system. 1 point is subtracted for early ischemic changes in the thalami, occipital cortex, and cerebellar hemispheres; and 2 points are subtracted if changes are present bilaterally. Additionally, 2 points are subtracted for early ischemic changes in the pons or midbrain. In a retrospective cohort of 130 patients evaluating the pc-ASPECTS, the sensitivity of the pc-ASPECTS for early ischemic changes was higher when calculated on the CTA-SI compared to the calculated score on the NECT in patients with suspected vertebrobasilar ischemia. Patients with basilar artery occlusion and a CTA-SI pc-ASPECTS of 7 or less had higher median baseline National Institute of Health Stroke Scale (NIHSS) scores (30 versus 13) and lower (14 versus 5) median baseline Glasgow Coma Scale (GCS) scores. CTA-SI pc-ASPECTSs were dichotomized from 0 to 7 and 8 to 10 to predict outcome. Patients with a score of 8–10 were more likely (RR, 12.1; 95% CI, 1.7–84.9) to have a good clinical outcome (mRS of 3 or less) compared to the 0–7 group. Additionally, a score of 7 or less was an independent predictor of mortality (RR, 0.4; 95% CI, 0.2–0.9) (7).

The pc-ASPECTS on CTA-SI was also evaluated in patients enrolled in Basilar Artery International Cooperation Study (BASICS). The BASICS is a prospective observational registry of patients with acute basilar artery occlusion. A CTA-SI pc-ASPECTS of 8 or more was related to reduced mortality (RR, 0.7; 95% CI, 0.5–0.98) and functional independence (RR, 2.0; 95% CI, 1.1–3.8) (8).

### Pons–Midbrain Index (PMI)

Another scoring scheme that places more emphasis on brainstem structures is the PMI. The CTA-SI is analyzed for hypoattenuation in the pons and midbrain. The pons and midbrain are bisected and each side is evaluated for hypoattenuation. A score of 0 implies no hypoattenuation; a score of 1 implies <50% hypoattenuation; and a score of 2 implies >50% hypoattenuation. Therefore, a score of 0 implies no hypoattenuation in the brainstem and a score of 8 implies >50% hypoattenuation bilaterally in the pons and midbrain.

In a retrospective review of the BASICS registry, a PMI <3 in comatose patients was associated with reduced mortality (adjusted RR, 0.66; 95% CI, 0.46–0.96) (9). In another retrospective study of 16 patients with vertebrobasilar occlusion treated with intra-arterial therapy (IAT), hypoattenuation evaluated in the pons on CTA-SI was the only independent predictor of outcome (10).

### pc-ASPECTS Score on DWI

**Figure 2:**
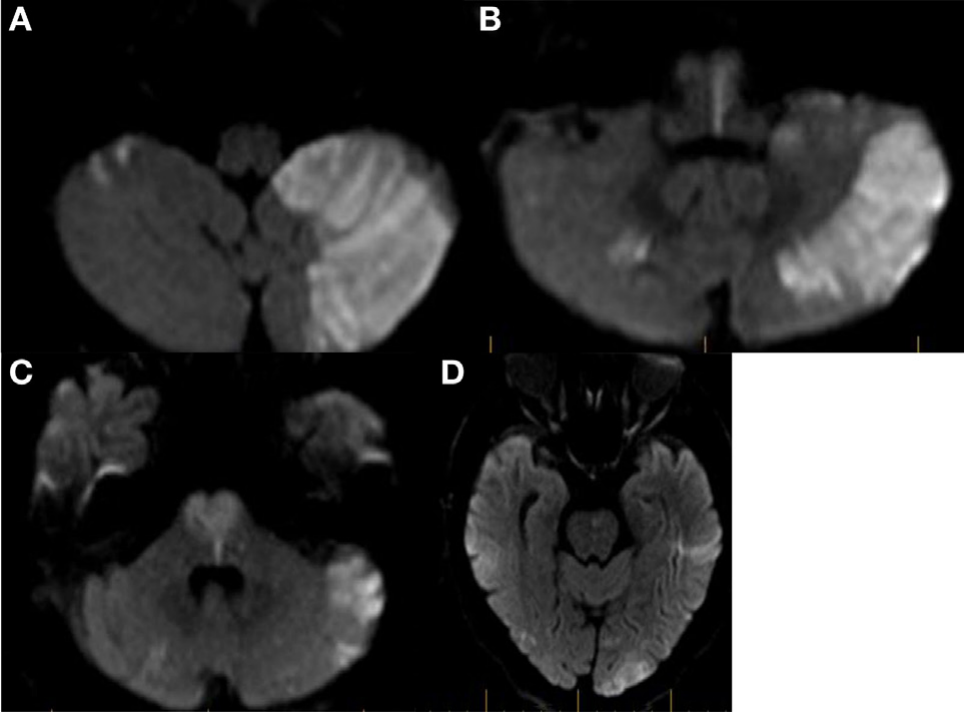
Example of Bern diffusion-weighted magnetic resonance imaging (DWI) and posterior circulation acute stroke prognosis early CT score (pc-ASPECTS) calculation. **(A)** Axial diffusion-weighted MRI in a patient with acute basilar artery occlusion. Restricted diffusion is seen in both cerebellar hemispheres. In the left cerebellar hemisphere >1/3 of the territory is involved. 3 points are assigned for the Bern DWI score and 2 points are assigned for the pcASPECTS score. **(B)** Restricted diffusion is noted involving bilateral pyramidal tracts at the level of the medulla. 4 points are assigned to the Bern DWI score and 0 points are assigned to the pc-ASPECTS. **(C)** Restricted diffusion is noted involving bilateral corticospinal tracts at the level of the pons. 4 points are assigned to the Bern DWI score and 2 points are assigned to the pcASPECTS score. **(D)** Restricted diffusion is noted in bilateral temporo-occipital regions. A score of 2 is assigned to both the Bern DWI score and the pcASPECTS score. Total Bern DWI score is 13. Total pcASPECTS score is 4.

The pc-ASPECTS may also be applied to DWI (Figure 2). In a retrospective study by Tei et al. of 132 patients with posterior circulation stroke, the pc-ASPECTS applied to DWI was an independent predictor of unfavorable functional outcome (OR, 0.40; 95% CI, 0.23–0.67) defined as an mRS of 3 or greater. The frequency of favorable outcome for patients with a DWI pc-ASPECTS of 5 or less was only 10% (11). In another retrospective study of 50 patients with acute basilar artery occlusion and early DWI, a DWI pc-ASPECTS of 8 or more was the only independent predictor for favorable outcome (OR, 3.9; 95% CI, 1.4–11.7) (12).

In a retrospective study of patients with basilar artery stenosis and vertebrobasilar ischemia, a poor DWI pc-ASPECTS was independently associated with early neurological deterioration and an unfavorable 90-day mRS (defined as an mRS of 3 or greater). Unlike other studies evaluating the DWI pc-ASPECTS, this study evaluated patients with basilar stenosis and not occlusion. A poor DWI pc-ASPECTS (defined as a DWI pc-ASPECTS of 6 or less) was an independent predictor of a worsening of the NIHSS by at least four points during the first 72 h after symptom onset (OR, 6.150; 95% CI, 2.872–13.170). Conversely, a DWI pc-ASPECTS of 7 or greater was an independent predictor of favorable 90-day outcome (mRS 0–2). This relationship persisted in the multivariate analysis of patients with and without early neurological deterioration. Interestingly, differences in treatment prior to or following early neurological deterioration did not predict neurological outcome (13).

### Renard DWI Score

Renard et al. developed a semiquantitative score based on the number of acute ischemic brain lesions on DWI. They prospectively studied 16 patients with acute basilar artery occlusion treated with IAT with recombinant tissue plasminogen activator. One point was assigned for unilateral involvement of brainstem structures or cerebellum and two points for bilateral involvement. Unilateral involvement of the thalamus or temporo-occipital lobe was assigned 5 points and bilateral involvement 1 point. In their univariate model, a lesion score of more than 3 was a predictor of poor outcome (mrS > 3) (14).

### Brainstem DWI (BS DWI) Score

Cho and colleagues described a semiquantitative score based on pretreatment DWI that predicted clinical outcome in 22 patients with basilar artery occlusion treated with endovascular intervention. Unlike the DWI pc-ASPECTS and the score proposed by Renard et al., the BS DWI score emphasizes arterial segmentation of the brainstem. The medulla and midbrain are divided into an anteromedial, anterolateral, lateral, and posterior segments and assigned 1 point for each arterial territory on each side for 8 possible points in either the medulla or midbrain. The pons is divided into an anteromedial, anterolateral, and lateral sections and assigned 1 point for each territory on each side for a maximum score of 6. Therefore, the total score may range from 0 to 22. In their univariate analysis, baseline NIHSS, BS DWI score, CS, and presence of coma were all statistically significant prognostic variables. In their multivariate regression analysis, only the BS DWI score was an independent predictor of neurological outcome (15).

### Bern DWI Score

Karameshev and colleagues used a combination of scoring systems proposed by Renard et al. and Cho et al. to develop an alternative scoring scheme known as the Bern DWI score (Figure 2). Unilateral lesions in the brainstem (including the medulla) are assigned one point, while bilateral lesions are assigned two points. If the corticospinal tract is affected, the assigned points are doubled. Therefore, a patient with restricted diffusion of bilateral corticospinal tracts in the pons would achieve a score of 4. Additionally, if <1/3 of the cerebellar hemisphere is involved, 1 and 2 points are assigned for unilateral and bilateral lesions, respectively. If the area of the cerebellar hemisphere involved is >1/3, the score is doubled. 1 or 2 additional points may also be assigned if the thalamus and temporo-occipital lobes are involved unilaterally or bilaterally. The Bern DWI score is especially notable for an emphasis on lesion localization, as double the points are assigned for corticospinal tract involvement, which is more likely to cause severe disability as measured by the mRS. Additionally, the score may double with larger hemispheric cerebellar infarctions that may be associated with cerebral edema and compression of the fourth ventricle. In a retrospective study of 36 patients comparing four different DWI-based scoring schematics (BS DWI, pc-ASPECTS, Bern DWI score, and Renard DWI score), all four scoring systems were significantly associated with functional outcome at 3 months in univariate analysis. When compared to the GCS score, the Bern DWI score was the only independent predictor of outcome that performed superior to the GCS in the multivariate analysis. A mean Bern DWI score of 8 ± 3 predicted a poor outcome or death (16).

### DWI Brainstem Score (DWI BSS)

Mourand and colleagues also described a DWI-based semiquantitative score that predicts clinical outcomes in patients undergoing mechanical thrombectomy with the Solitaire FR Device for acute basilar artery occlusion. The DWI BSS like the BS DWI score divides and hemisects the brainstem. One point is assigned for each unilateral high-signal intensity lesion that occupies less than half of the area and 2 points are assigned if the lesion occupies more than half the area. The score ranges from 0 to 12. A DWI BSS of <3 was an independent predictor (OR, 9.92; 95% CI, 1.75–56.30) of favorable outcome (mRS of 2 or less) and a DWI BSS of 6 or greater had a 100% specificity for poor outcome (mRS of 5 or greater) (17).

## Extent of Collateral Flow

### Posterior Circulation Collateral Score (PC-CS)

Posterior circulation collateral flow has also been evaluated to predict clinical outcome in acute basilar artery occlusion. The primary collateral pathway of the posterior circulation from the anterior circulation occurs *via* the posterior communicating artery (PCOM). Prior studies using catheter cerebral angiography have confirmed the favorable outcome in patients with collateral filling of basilar artery (18).

Collateral pathways may also be evaluated *via* CTA. In a retrospective study of patients enrolled in the BASICS registry, Hoeven et al. developed a 10-point score to quantify collateral flow in the PCOMs and the cerebellar arteries. Each patent cerebellar artery [posterior inferior cerebellar artery (PICA), anterior inferior cerebellar artery (AICA), and superior cerebellar artery (SCA)] is allocated 1 point for each side. Each patent PCOM is allocated 1 point if the diameter is smaller than the ipsilateral P1 segment of the posterior cerebral artery (PCA) and 2 points if the diameter is larger than the ipsilateral P1 segment of the PCA. The PC-CS was trichotomized into poor (0–3), intermediate (4–5), and good (6–10) collateral scores (CSs). Patients with a poor PC-CS more often had a severe deficit (coma, tetraplegia, or locked-in-state) compared to patients with an intermediate or good PC-CS. When a good score was compared to a poor score, there was a statistically significant lower risk (RR, 0.74; 95% CI, 0.58–0.96) for poor outcome (mRS of 4 or greater). Additionally, the presence of 1 or more patent PCOM was associated with a statistically significant lower risk (RR, 0.79; 95% CI, 0.66–0.95) for poor outcome. If at least one PCOM was larger caliber than the ipsilateral P-1 segment of the PCA, then the risk of a poor outcome was lower in the multivariate analysis (RR, 0.76; 95% CI, 0.61–0.96) (19).

### Collateral Score (CS)

Another retrospective study specifically evaluated the importance of PCOM collaterals. Goyal and colleagues assigned a pretreatment CS based only on the presence or absence of PCOMs. A score of 0 is assigned in the absence of bilateral PCOMs. A score of 1 to the presence of 1 PCOM and a score of 2 for the presence of bilateral PCOMs. Scores were dichotomized into poor (0 or 1) or good (2). Compared to patients with a poor CS, patient with a good CS were more likely to have a favorable outcome and a decrease in the NIHSS during hospitalization. Improved outcome measures, however, were not confirmed in their multivariate regression models (20).

## Thrombus Burden and Location

### Posterior Circulation Computed Tomography Angiography (pc-CTA) Vascular Score

Ros and colleagues created a CTA-based score based on the extent of arterial occlusion. The vertebrobasilar tree was divided into six segments and absence of flow was assigned 1 point. Points were assigned as follows: 1 point for either intracranial vertebral artery; 1 point for the proximal segment of the basilar artery (defined as the basilar artery origin to the origin of AICA); 1 point for the middle segment of the basilar artery (defined as the segment between AICA and SCA); and 1 point for the distal segment of the basilar artery (defined as the segment between the SCA and P-1 segment of the PCA); and 1 point for each PCA. Therefore, a score of 0 implies complete patency and a score of 6 implies occlusion of the posterior circulation arterial tree. Patients with a favorable outcome (mRS of 3 or lower) had statistically significant lower pc-CTA scores (2; interquartile 25–75%: 1–2) compared with patients in the poor outcome group (3: interquartile 25–75% 3–4) (21).

### Basilar Artery on Computed Tomography Angiography (BATMAN) Score

**Figure 3:**
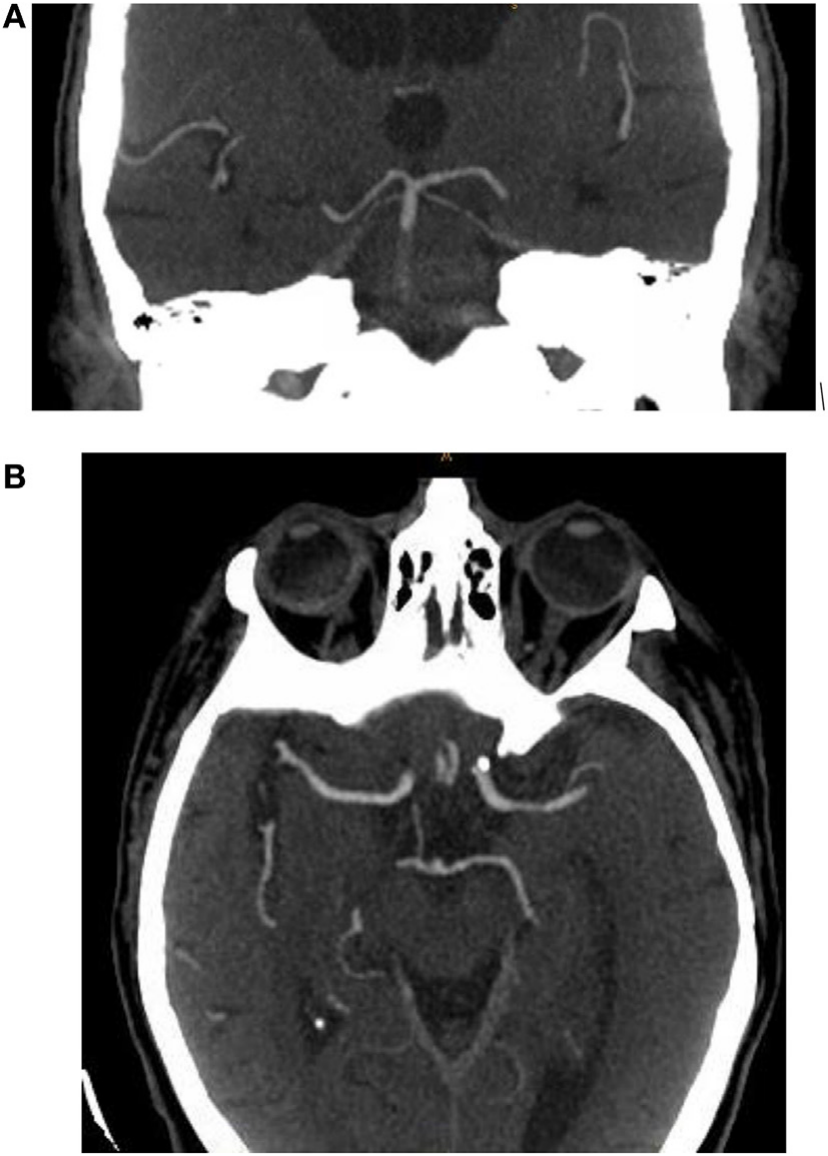
Example of basilar artery on computed tomography angiography (BATMAN) score calculation. Coronal maximum intensity projection of a computed tomography angiogram in a patient with acute basilar artery occlusion. **(A)** 1 point is assigned to normal arterial filling of the distal segment of the basilar artery and 2 points for normal arterial filling of bilateral P-1 segments of the posterior cerebral artery. **(B)** Axial maximum intensity projection of a computed tomography angiogram in the same patient. 2 points are assigned to normal arterial filling in the right posterior communicating artery. Total BATMAN score is 5.

Alemseged and colleagues described another CTA scoring system, the BATMAN score, similar to the pc-CTA score (Figure 3). The scoring system is based on presence of arterial filling on CTA and points are assigned *via* the following: 1 point for both vertebral arteries (considered as 1 segment); 1 point for the proximal segment of the basilar artery; 1 point for the middle segment of the basilar artery; 1 point for the distal segment of the basilar artery; 1 point for the presence of each P-1 segment of the PCA; and 2 points for filling of each PCOM. 1 point is awarded instead of two for filling in a hypoplastic PCOM. Therefore, a score of 10 suggests normal arterial filling of the posterior circulation and a score of 0 suggests no filling of the posterior circulation. In their multivariate analysis, an unfavorable BATMAN score (defined as <7) was independently associated with poor outcome (defined as an mRS of 4 or greater) in both derivation and validation cohorts. A BATMAN score of <7 had a sensitivity of 84% and specificity of 76% to predict a poor outcome (22).

### FLAIR-Hyperintense Basilar Artery (FHBA) Sign

**Figure 4:**
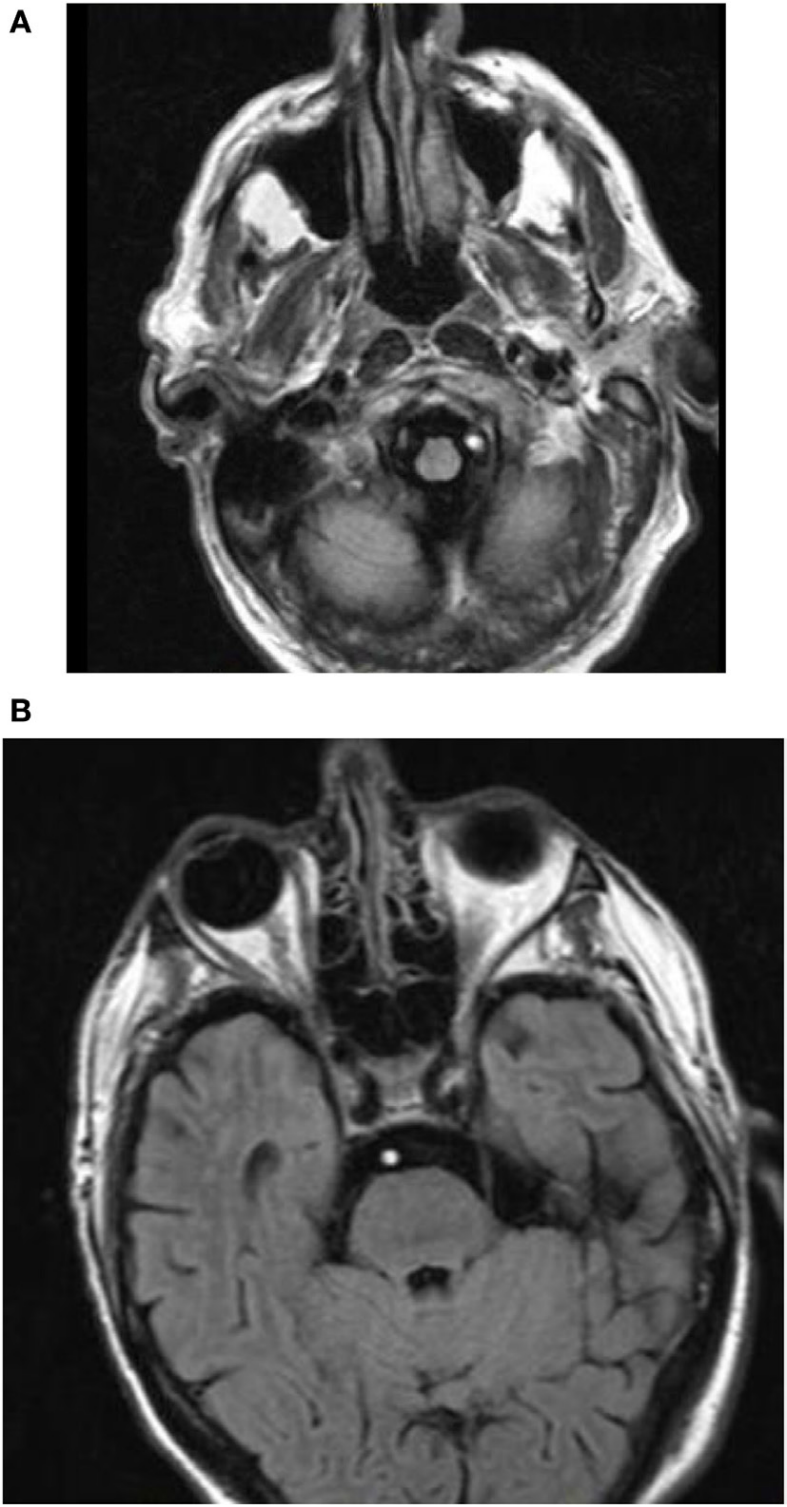
Fluid-attenuated inversion recovery-hyperintense vessel. **(A)** Hyperintense signal noted in the left vertebral artery in a patient with acute basilar artery occlusion. **(B)** Contiguous hyperintense signal noted in the basilar artery in the prepontine cistern in the same patient.

Gawlitza and colleagues retrospectively evaluated the hyperintense configuration of the basilar artery on the fluid-attenuated inversion recovery (FLAIR) MRI sequence in 20 patients with acute basilar artery occlusion to predict clinical outcome (Figure 4). FLAIR-hyperintense vessels are most likely caused by both impaired blood flow and intrinsic signal intensity of the thrombus. In patients with a FHBA sign, a score was generated by “counting the number of sections from the basilar tip down to the foramen magnum, including the dominant vertebral artery, in which a hyperintense signal in the vessel lumen was present multiplied by section thickness.” When dichotomized to favorable (mRS of 2 or less) or unfavorable (mRS of 3 or more) outcomes, there was no significant difference among the mean FHBA sign scores between the groups. There was, however, a statistically significant association between higher scores and death. A FHBA sign score of greater than or equal to 17.5 yielded a sensitivity of 83% and specificity of 77% for mortality. Occlusion site was also found to be a predictor of patient outcome. All patients with occlusion of the proximal basilar artery died, compared to 82% survival rates with occlusion in the mid-basilar section and 100% survival with thrombus at the basilar tip (23).

## Discussion

Both clinical and imaging findings may independently predict clinical outcome in patients with acute basilar artery occlusion (Table 1). Multiple imaging-based scoring schematics support the use of imaging to accurately compare outcome in patients with acute basilar artery occlusion. This is especially applicable to future randomized controlled trials and observational registries that compare treatment strategy.

**Table 1:** Radiographic versus clinical predictors of poor outcome in acute basilar artery occlusion.

Radiographic predictors of poor outcome in acute basilar artery occlusion	Clinical predictors of poor outcome in acute basilar artery occlusion
No recanalization	Age
HDBA sign on NCCT	Higher admission NIHSS
pc-ASPECTS of 7 or less on computed tomography angiogram source images	Lower GCS on presentation
pc-ASPECTS of 7 or less on DWI	Diabetes mellitus
DWI BSS of 6 or greater	Hypertension
Proximal FHBA sign	
FHBA sign of 17.5 or greater	
BATMAN score of <7	
Bern DWI score 8 ± 3 (SD)	

*HDBA, hyperdense basilar artery; pc-ASPECTS, posterior circulation acute stroke prognosis early CT score; DWI, diffusion-weighted image; BSS, brainstem score; FHBA, FLAIR hyperintense basilar artery; NIHSS, National Institute of Health Stroke Scale; GCS, glasgow coma scale; BATMAN, basilar artery on computed tomography angiography*.

For example, in the large prospective observational BASICS registry, there was no statistically significant superiority for any treatment strategy (24). Although baseline characteristics for treatment type included measures of clinical severity, the location of the occlusion was the only radiographic characteristic included in the analysis. The lack of baseline non-invasive cranial imaging may confound the conclusion of this study. Baseline imaging parameters on non-invasive cranial imaging was also superior to onset to treatment time for the prediction of clinical outcome in one prospective observational study (25) and another prospective and retrospective registry study (26). These studies support the importance of baseline non-invasive cranial imaging in future studies.

Scoring schematics are primarily grouped in those that evaluate for early ischemic changes, the extent of collateral circulation, thrombus location, and thrombus burden. CTA-SI and DWI may both evaluate early ischemic changes. Scoring schematics that place a special emphasis on early involvement of the brainstem corticospinal tracts and large cerebellar infarctions may be more reliable than others at predicting outcome. The comparison study by Karameshev et al. is suggestive of this. Involvement of the corticospinal tracts may lead to more functional deficits compared to the structures in the lateral brainstem. Additionally, the presence of large hemispheric cerebellar infarctions may cause cerebral edema and compression of the fourth ventricle. Therefore, large cerebellar lesions are also likely to favor a poor clinical outcome independent of brainstem status.

Although early ischemic changes on DWI may be more sensitive to detect early ischemic changes compared to CTA-SI, CTA is a much more readily available imaging study, especially in the urgent evaluation of ischemic stroke. A limitation in the evaluation of early ischemic changes on CTA-SI is the dependence on acquisition protocols. In a retrospective study by Pulli et al., a shorter time between contrast material injection and image acquisition was an independent predictor of infarct size overestimation on CTA-SI when compared to DWI (27). These findings were consistent in another retrospective study by Yoo and colleagues (28). This may be problematic in future multicenter trials comparing the CTA-SI pc-ASPECTS between groups if the CTA acquisition protocol is not standardized. In the retrospective study by Ros et al., they noted evaluation of the CTA-SI pc-ASPECTS to be “technically challenging with both a low interobserver concordance and poor correlation with clinical outcome.” More data are necessary to determine if infarction overestimation on CTA-SI meaningfully changes its prognostic value.

Other scoring schematics have focused on the extent of collateral flow. The ENDOSTROKE multicenter registry enrolled 148 patients with angiographically proven basilar artery occlusion. In their multivariate analysis, collateral status as graded by the American Society of Interventional and Therapeutic Neuroradiology/Society of Interventional Radiology (ASITN/SIR) collateral grade was an independent predictor of both outcome and recanalization (26). The ASITN/SIR collateral grade is based on time of collateral filling and extent of collateral flow in the ischemic territory and cannot be replicated by current non-invasive cranial imaging.

Current CTA-based scoring schematics have primarily focused on the PCOM. The PCOM is the major collateral pathway between the anterior and posterior circulations. This collateral pathway may only be beneficial if a thrombus does not fully obstruct the basilar tip or if it does not extend from the basilar tip into the P-1 segment as retrograde filling of the basilar artery would be impaired in both situations. In a retrospective study of patients with acute basilar artery thrombosis treated with intra-arterial urokinase, there was no correlation found between the number of PCOMs (evaluated by catheter cerebral angiography) and symptom duration, pretreatment GCS score, survival, or neurological outcome (18). Therefore, a scoring schematic, which only evaluates the presence or absence of a PCOM may underscore the importance of thrombus burden.

Thrombus location may also play an important role in predicting clinical outcome. Two studies showed that proximal basilar occlusions are more likely unfavorable predictors of outcome compared to distal occlusions (13, 29). This is consistent with a prior catheter cerebral angiography-based study of 22 patients who evaluated predictors of survival and good neurological outcome in patients treated with basilar artery thrombolysis. The study found that the single best predictor of survival of patients was distal clot location (30). The authors suspected that distal emboli are more likely embolic in origin and may have a more favorable response to lysis compared to thrombus formation at the site of atherosclerosis often found in the mid-basilar and proximal segments. Additionally, a thrombus may be more prone to recur in the setting of local atherosclerosis.

Conversely, in the study by Ros et al., patients with involvement of the distal segments of the basilar artery had the worst outcome. They note that in the setting of complete occlusion of the distal basilar artery, there may be limited retrograde flow from the PCOM. Additionally, patients with proximal occlusion are more likely to have antecedent stenosis and develop more robust collateral circulation. They also note that brainstem paramedian perforators are more quickly occluded in basilar apex syndromes due to “back thrombosis.”

In the ENDOSTROKE registry, thrombus location did not predict outcome (26). More data are necessary to determine if thrombus location is an independent predictor of clinical outcome.

Thrombus burden is a promising way to predict clinical outcome as shown in the studies by Ros et al. and Alemseged et al. The pc-CTA and BATMAN scores are analogous to the Clot Burden Score for anterior circulation large vessel occlusions. The major difference between the pc-CTA and BATMAN scores are the addition of points assigned to the PCOM in the BATMAN score. This modification takes into account collateral flow; therefore, the BATMAN score is a reflection of both thrombus burden and collateral flow. The authors of the proposed BATMAN score note that absence of PCOMs was the strongest predictor of poor clinical outcome and, therefore, was weighted higher compared to other arterial segments.

Several major limitations exist with the literature regarding neuroimaging predictors of clinical outcome in acute basilar artery occlusion. Many of the scoring schematics were developed with a small number of patients. Additionally, comparing and contrasting different schematics is challenging due to different treatments modalities and different definitions of good clinical outcome. Finally, although many of the scoring schematics had excellent intraobserver and interobserver reliability, the BATMAN score was the only score that was externally validated. Lack of external validation limits the generalizability of other scoring schematics.

## Conclusion

Several of the scoring schematics described were predictors of clinical outcome independent of recanalization status and, therefore, future studies may benefit from including more radiographic characteristics. Additional data are needed to further compare and contrast scoring schema used for acute basilar artery occlusion.

## Author Contributions

Research and drafting the article: RG. Critically revising the article: JB.

## Conflict of Interest Statement

The authors declare that the research was conducted in the absence of any commercial or financial relationships that could be construed as a potential conflict of interest.
